# Gene Expression-Based Chemical Genomics Identifies Potential Therapeutic Drugs in Hepatocellular Carcinoma

**DOI:** 10.1371/journal.pone.0027186

**Published:** 2011-11-07

**Authors:** Ming-Huang Chen, Wu-Lung R. Yang, Kuan-Ting Lin, Chia-Hung Liu, Yu-Wen Liu, Kai-Wen Huang, Peter Mu-Hsin Chang, Jin-Mei Lai, Chun-Nan Hsu, Kun-Mao Chao, Cheng-Yan Kao, Chi-Ying F. Huang

**Affiliations:** 1 Institute of Clinical Medicine, National Yang-Ming University, Taipei, Taiwan; 2 Department of Computer Science and Information Engineering, National Taiwan University, Taipei, Taiwan; 3 Institute of Biomedical Informatics, National Yang-Ming University, Taipei, Taiwan; 4 Institute of Information Science, Academia Sinica, Taipei, Taiwan; 5 Graduate Institute of Biomedical Electronic and Bioinformatics, National Taiwan University, Taipei, Taiwan; 6 Department of Surgery & Hepatitis Research Center, National Taiwan University Hospital, National Taiwan University, Taipei, Taiwan; 7 Department of Life Science, Fu-Jen Catholic University, New Taipei City, Taiwan; 8 Information Sciences Institute, University of Southern California, Marina del Rey, California, United States of America; 9 Institute of Biopharmaceutical Sciences, National Yang-Ming University, Taipei, Taiwan; Florida International University, United States of America

## Abstract

Hepatocellular carcinoma (HCC) is an aggressive tumor with a poor prognosis. Currently, only sorafenib is approved by the FDA for advanced HCC treatment; therefore, there is an urgent need to discover candidate therapeutic drugs for HCC. We hypothesized that if a drug signature could reverse, at least in part, the gene expression signature of HCC, it might have the potential to inhibit HCC-related pathways and thereby treat HCC. To test this hypothesis, we first built an integrative platform, the “Encyclopedia of Hepatocellular Carcinoma genes Online 2”, dubbed EHCO2, to systematically collect, organize and compare the publicly available data from HCC studies. The resulting collection includes a total of 4,020 genes. To systematically query the Connectivity Map (CMap), which includes 6,100 drug-mediated expression profiles, we further designed various gene signature selection and enrichment methods, including a randomization technique, majority vote, and clique analysis. Subsequently, 28 out of 50 prioritized drugs, including tanespimycin, trichostatin A, thioguanosine, and several anti-psychotic drugs with anti-tumor activities, were validated via MTT cell viability assays and clonogenic assays in HCC cell lines. To accelerate their future clinical use, possibly through drug-repurposing, we selected two well-established drugs to test in mice, chlorpromazine and trifluoperazine. Both drugs inhibited orthotopic liver tumor growth. In conclusion, we successfully discovered and validated existing drugs for potential HCC therapeutic use with the pipeline of Connectivity Map analysis and lab verification, thereby suggesting the usefulness of this procedure to accelerate drug repurposing for HCC treatment.

## Introduction

Hepatocellular carcinoma (HCC) is the most common liver malignancy and one of the leading causes of cancer death worldwide. It is an aggressive tumor, and the median survival period following diagnosis is approximately 6 to 20 months [1]. Surgical resection is the main form of therapy; however, the majority of patients are not resectable due to the late stage of the disease or poor liver preservation. Liver transplantation, radiofrequency ablation, percutaneous ethanol ablation, transarterial chemoembolization, and targeted therapy are other standard treatments. Currently, only sorafenib has been approved by the FDA for HCC treatment [2], [3]; however, in the phase III, double-blind, placebo-controlled trial, the median overall survival period in the sorafenib group was prolonged by only 2.8 months compared with the placebo group [3]. Therefore, there is great urgency to identify additional drugs for treating HCC (see review [4]).

Several studies [5], [6] have utilized a novel technique to discover potentially therapeutic chemicals through a collection of chemically-induced gene expression profiles. This method includes searching for anti-correlated expression patterns of the genes of interest. Through proof of concept studies, the “Connectivity Map” (CMap) project was developed to host a much greater number of gene-expression profiles from cultured human cancer cell lines treated with bioactive small molecules and to provide pattern-matching algorithms to mine these data [7]. The platform-independent system uses a nonparametric, rank-based algorithm to calculate a score that indicates the degree of similarity or dissimilarity between the query gene signatures and profile gene signatures. A strong positive connectivity score (similarities) indicates that the corresponding agent of that profile induces the expression of the query, while a strong negative connectivity score (dissimilarities) shows that the corresponding agent reverses the expression of it. Thus, agents with strong negative connectivity scores might drive a particular disease state into a more stable state [8], [9]. The use of non-parametric statistics enables users to compare signatures across various array platforms without resorting to complicated meta-analysis. The flexible system offers an opportunity to identify potential drugs targeting specific diseases. For example, all-trans retinoid acid (ATRA) in acute promyelocytic leukemia and imatinib in gastrointestinal stromal tumors and chronic myeloid leukemia have been shown to target the specific pathways associated with the type of carcinogenesis and have good treatment outcomes [10], [11].

In the post-genomics era, advances in tools (CMap) and microarray profiling have provided an excellent opportunity to monitor global gene expression in HCC [12]–[15] and to better understand the complex interactions of hepatocarcinogenesis. These high-throughput analyses have identified numerous differentially expressed genes and have aided in the identification of disease markers for diagnosis and potential targets for treatment. To better embrace the paradigm shift, we have improved our information-harvesting infrastructure, the Encyclopedia of Hepatocellular Carcinoma genes Online 2, dubbed EHCO2 (http://ehco.iis.sinica.edu.tw). EHCO2 employs natural language processing and softbots (or Web wrapper agents [16]) to collect scattered gene annotations either by mining data sources directly or by querying publicly accessible databases.

Since the etiology of HCC (i.e., HCV, HBV, or alcohol-related) differs in its molecular carcinogenesis, the ultimate aim is to discover drugs that exploit either distinct etiology-related targets or common targets. With no proper array paired samples or ample clinical data (Table 1) to separate subgroups in the EHCO2 data, however, we can only concentrate on the latter aim. Despite these limitations, various gene signature selection methods were employed to identify common genetic signatures. Using these HCC gene signatures and the CMap tool, a combination of computational and experimental studies identified several potential therapeutic drugs for the treatment of human HCC.

**Table 1:** HCC set criteria and individual gene counts.

Group	Name	Number of up/downregulated genes	Sample Size	Features	Selection Criteria
1	SMD	90/180	102 primary HCC and 74 non-tumor tissues	HBV, HCV	Intersected with STITCH[19]
	GIS	160/38	37 HBV and non-tumor tissues (paired)	HBV	
	LEE_NIH	161/153	91 human HCC and 7 mouse HCC	Mouse vs human models	
	KIM_NIH	46/178	59 cirrhotic tissues, 14 HCC	HBV, HCV	(mixed signature)
	CGED	305/291	120 HCC tissues, 86 non-tumor tissues and 32 normal liver tissue		
	FUDAN	201/292	29 HCC and 29 non-tumor tissues (paired)	HBV	
	PASTEUR	31/53	15 HCC, 15 non-tumor tissues (paired)	HBV, HCV	(mixed signature)
	TOKYO	94/147	20 HCC and 20 non-tumor tissues (paired)	HBV, HCV	(mixed signatures)
	SMD_3K	8/15			Gens with 3-cliques
	CGED_3K	12/9			Gens with 3-cliques
	FUDAN_3K	13/10			Gens with 3-cliques
2	250-gene sets(100 sets)	250/250			Randomly selected from EHCO2
	500-gene sets(100 sets)	500/500			Randomly selected from EHCO2
	1000-gene sets(100 sets)	1000/1000			Randomly selected from EHCO2
	Frequent Sets(494 sets)				Derived from Confident set
	Clique Sets(256 sets)				

## Materials and Methods

### Ethics Statement

All animal experiments were performed in accordance with the guidelines of the Animal Welfare Committee of National Taiwan University College of Medicine. (Approval ID: 20090352)

### Collection of HCC-related gene expression signatures

**Figure 1:**
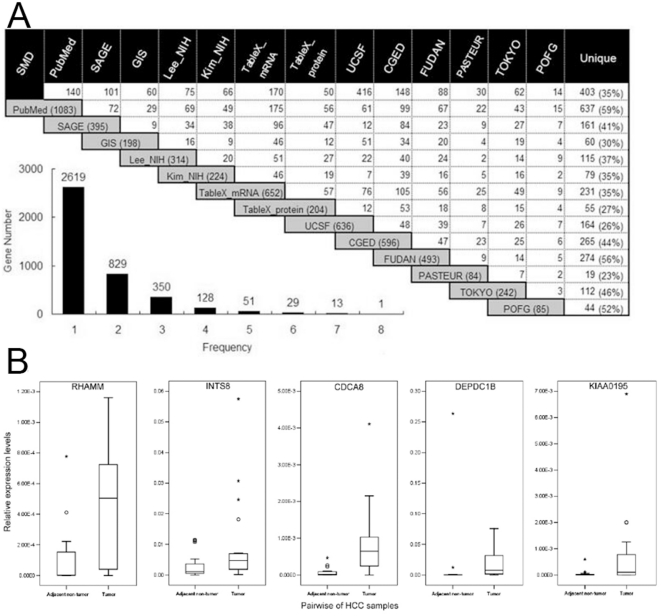
Collection, intersection, and validation of HCC-related genes in EHCO2. (**A**) **Gene sets in EHCO2 and their intersecting genes.** The gray box indicates the number of genes reported in each set, while the intersection cell indicates the numbers of common genes. Each pair of datasets shares a small number of common genes, suggesting the heterogeneous nature of HCC. The bottom-left insert shows the frequency of genes reported. Most genes are reported only once. (**B**) **Validation of up-regulated genes via Q-RT-PCR.** RHAMM, INTS8, CDCA8, DEPDC1B, and KIAA0195 are over-expressed in 21 paired HCC patient samples.

A fundamental part of EHCO2 was the collection of 14 HCC-related gene sets from PubMed as well as diverse high-throughput studies [17], computational predictions, and validations [18] (Figure 1A). The details of each set are listed in Table 1 and Method S1.

### Validation of EHCO2 genes by Q-RT-PCR

The mRNA expression levels were determined by quantitative RT-PCR in 21 pairs of HCC patients (from the Taiwan Liver Cancer Network, see Acknowledgement). The results were normalized to the mRNA expression level of GAPDH in each sample (Figure 1B).

### Generation of HCC test sets

Two groups of datasets were used in this study; the details are summarized in Table 1. Group 1 contained the original eight sets of HCC gene signatures derived from EHCO2 microarray-based studies. Group 2 contained sets derived from Group 1, including randomized sets, sets derived from combinations of studies, and sets from Clique Analysis.

#### a) Generation of Group 1: Original EHCO2 sets

Group 1 contained the original eight sets of microarray-based HCC gene expression profiles from EHCO. The other six sets contained no microarray information and, thus, were excluded from further analysis. The UCSF and POFG sets were discarded since they only contained up-regulated genes. The SMD set, in which the number of differentially expressed probe sets exceeded CMap's limit of 1,000 probe sets, was filtered using the STITCH [19] database such that all genes had known interacting proteins.

#### b) Generation of Group 2: Derived EHCO2 sets

The Group 2 datasets were derived from the Group 1 data. The set, “100 random sets,” was generated to reflect a variety of HCC conditions, using a randomization technique to simulate possible combinations. The Confident Set (Method S1) was used as the pool for the randomization. Only genes with Affymetrix U133A annotations were retained, resulting in a smaller set of 1,588 up-regulated and 1,308 down-regulated genes. The set consisted of 100 sets of 250 randomly selected up-regulated genes and 250 randomly selected down-regulated genes. The randomly selected genes were converted into the probe IDs of the Affymetrix U133A platform by using the R packages from BioConductor [20]. In addition, to be able to closely represent the complete HCC conditions, sets using 500 up-regulated and 500 down-regulated genes and sets using 1,000 up-regulated and 1,000 down-regulated genes were also generated. To efficiently conduct massive calculations, a program written in Ruby implemented the CMap core algorithm and utilized CMap's original data. The results were compared to Cmap's output and verified for exactness. The program also overcame the CMap input limit of 1,000 probe sets, making it feasible to run sets with larger input size.

Furthermore, two sets were generated to enrich the HCC gene expression profile. The “Frequent sets” were created using all combinations (n) of eight EHCO2 sets and three frequency thresholds (k). For instance, for n = 7, or C^8^_7_, eight sets were created, each with one set omitted; for k = 2, genes presented 2 times or more were included. This criterion extracted the most common altered HCC genes for further testing. In addition, to further enrich the gene sets, Clique Analysis [21] was employed. The term *clique*, originating from the field of Graph Theory, describes nodes of a sub-graph that have connections to all the other nodes in that sub-graph. For example, a 3-clique is a graph with 3 interconnected nodes, which is also a triangle. The genes were used to construct their Protein-Protein Interaction (PPI) network, where we were able to make calculations to select proteins with complete interactions. The “Clique sets” contained all “Frequent sets” that had undergone Clique Analysis.

### CMap Analysis

**Figure 2:**
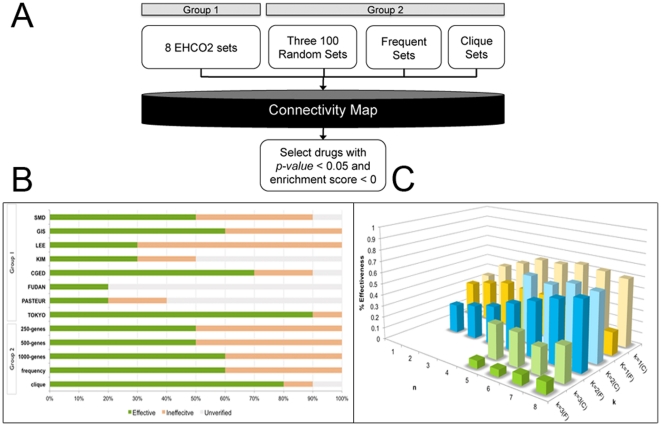
Flowchart and comparisons of prediction accuracy. (**A**) **The CMap analysis flowchart.** All eight sets from EHCO2 (Group 1), 100-member random sets, Frequent sets, and Clique sets (Group 2) were individually queried with CMap. Only drugs with a *p-value* of less than 0.05 and a negative enrichment score were retained. (**B**) **Comparison of the accuracy of predicted drugs from each set.** The top 10 drugs from each set were labeled according to their anti-cancer effects. (**C**) **The comparison of the Frequency sets and the Clique sets.** The average effectiveness of drugs was compared side by side.

The CMap analysis steps are illustrated in Figure 2A. Each set, consisting of up- and down-regulated genes, was input into CMap according to the program's instructions. Sets with less than 10 up-regulated (or down-regulated) probe sets were discarded due to limited input. Only drugs with negative scores and *p-values* of less than 0.05 were retained. Drug occurrences were summed and used to rank the drugs.

### Chemicals, cell culture, MTT cell viability assay and clonogenic assay

The HCC cell lines, Mahlavu, PLC5, HepG2, and Huh7, were cultured in Dulbecco's Modified Eagle Medium (DMEM; Seromed, Berlin, Germany) supplemented with 10% heat-inactivated fetal bovine serum, 100 µg/ml streptomycin, 100 µg/ml penicillin, and 2 mM L-glutamine in a humidified atmosphere containing 5% CO_2_ at 37°C. The viability of the exposed cells was determined using the MTT cell viability assay kit (Sigma-Aldrich, St. Louis, USA), according to the manufacturer's instructions. The tetrazolium salt 3-[4,5-dimethylthiazol-2-yl]-2,5- diphenyltetrazolium bromide (MTT) is used to determine cell viability in assays of cell proliferation and cytotoxicity. Twenty-four hours after seeding cells at a concentration of 1.5×10^3^ cells/well in 100 µl of culture medium in a 96-well microplate, the cells were then treated with small molecules (Table 2) selected from the drug lists from the CMap queried results. The cells were exposed to different concentrations of the small molecules for 72 hours. Control cells were incubated in the absence of small molecules. Afterwards, the cells were incubated with medium containing MTT for 2 hours. The optical density at 450 nm was measured using a microplate reader (Spectral Max250). For the clonogenic assay, Huh7 cells were seeded out in appropriate dilutions in a 6-well plate and treated with selected small molecules at various concentrations for 15 days. Colonies were fixed with glutaraldehyde (6.0% v/v), stained with crystal violet (0.5% w/v), and counted.

**Table 2:** The most frequently identified drugs from the Top 20 drugs of Group 1 (EHCO2 sets) and Group 2 (Derived EHCO2 sets).

Drug Name	Description	Frequency*	IC50(µM)	ClonogenicAssay**	PubMedcancer	PubMedHCC
Tanespimycin	HSP90 inhibitor	3	<0.1	N/A	Yes[24]	Yes[24]
Trichostatin A	HDAC inhibitor	9	0.1∼1	N/A	Yes[25],[26]	Yes[32]
Thioguanosine	Purine analog	9	5∼10	N/A	Yes[27]	Yes[27]
Thioridazine	Antipsychotic drugs	4	5∼10	N/A	Yes[37]	No
Phenoxybenzamine	Antihypertensive drugs	6	>10	Effective	No	No
Trifluoperazine	Antipsychotic drugs	2	>10	Effective	Yes[37]	No
Dipyridamole	Platelet aggregation inhibitor	5	>10	Effective	Yes[37]	No
Sulconazole	Antifungal agents	7	>10	Effective	No	No
Apigenin	Flavone	8	>10	Effective	Yes[37]	Yes[37]
Chlorpromazine	Antipsychotic drugs	4	>10	Effective	Yes[36],[38]	No
Luteolin	Flavonoid	9	>10	Effective	Yes[39]	Yes[39]
Medrysone	Steroid	9	>10	Ineffective	No	No
8-azaguanine	Purine analog	7	>10	Ineffective	Yes[40]	No
Repaglinide	Antidiabetic Agents	7	>10	Ineffective	No	No
Alpha-estradiol	Hormone	7	>10	Ineffective	No	No

*: The frequency of drugs appeared in top 20 drugs in group 1 and group 2.

**: Effectiveness in the clonogenic assay is defined as reducing the number of colonies by more than 50% at 10 µM. Table is sorted by IC_50_ value.

### Animal study

The mouse hepatoma cell line BNL was purchased from the American Type Culture Collection (Rockville, MD) and maintained in DMEM supplemented with 10% fetal calf serum. Male BALB/c mice aged 7–8 weeks were used in the experiments. All animal experiments were performed in accordance with the guidelines of the Animal Welfare Committee of National Taiwan University College of Medicine. For the single HCC nodule model, 3×10^5^ BNL cells were injected into the left liver lobe of mice on day 0. The needle hole was sealed with an electric coagulator (Aaron, Petersburg, Florida, USA) immediately after the withdrawal of the needle to avoid leakage. The incision was subsequently sutured. In the prophylactic experiment, chlorpromazine (10 mg/kg/day) or trifluoperazine (10 mg/kg/day) were administered orally beginning on day 1 after tumor formation (n = 10 for each group); in the therapeutic experiment, these test agents were administered beginning on day 14. The tumors were measured using calipers after the 21-day treatment by an investigator blinded to the treatment groups. The tumor volume was calculated using the following formula: volume = width^2^×length×0.52.

## Results

### Generation of EHCO2 data

To have a more thorough collection of HCC-related gene expression profiles, EHCO2 was expanded from eight gene-set collections to 14 gene-set collections, totaling 4,020 non-redundant genes (the additional six gene-sets collections are described in Method S1). Figure 1A shows the intersection between each gene set. The SMD and UCSF datasets had the greatest overlap of 416 genes. Interestingly, 35% of the SMD (403 out of 1,160) and 26% of the UCSF (164 out of 636) collections (referring to distinct genes in Figure 1A) were genes that have not been reported in other gene sets. A cross-dataset comparison of 14 datasets revealed the 14 genes that were most often identified, which appeared at least seven times each in EHCO2 (Figure 1A). However, the majority (∼65%) of genes in the EHCO2 collections (see the bar chart in Figure 1A) appeared only once, and there were some discrepancies among the gene sets, indicating the need for further validation. Thus, we randomly selected five genes that had an “Up” expression pattern in EHCO2 for validation of their expression using quantitative RT-PCR. As shown in Figure 1B, RHAMM, INTS8, CDCA8, DEPDC1B, and KIAA0195 are over-expressed in 21 of the paired HCC patient samples examined.

To shed new light on the *in silico* drug-screening platform CMap, EHCO2 data were used for creating gene signatures. Since the utility of CMap relies on its use of non-parametric statistics, no meta-analysis was required when combining data from various sources, making it manageable to conduct studies with data from different array platforms. To utilize CMap, two groups of gene signatures were created from the EHCO2 database to allow a comparison of the results for the best prediction power.

### Gene signatures and CMap analysis of Group 1 sets (original EHCO2 sets)

Group 1 contained the original eight microarray-based HCC gene expression profiles from EHCO2 (Table 1), with an average of 136 up-regulated and 166 down-regulated genes. Before the CMap analysis, the degree of data consistency was analyzed using Jaccard's Index (Method S1) as a measure of set similarity (Table S1). Figure S1 shows that each set had a very high distance from (or low similarity to) each other based on the clustering result using Jaccard's distance (i.e., one minus Jaccard's Index) as a dissimilarity measure. Even though sets marked as up-regulated were ideally separated from those marked as down-regulated, the up-regulated KIM set showed very little resemblance to the others. Similar to other studies [22], our analysis showed the heterogeneous nature of HCC, indicating that HCC may comprise multiple states and/or subtypes.

**Figure 3:**
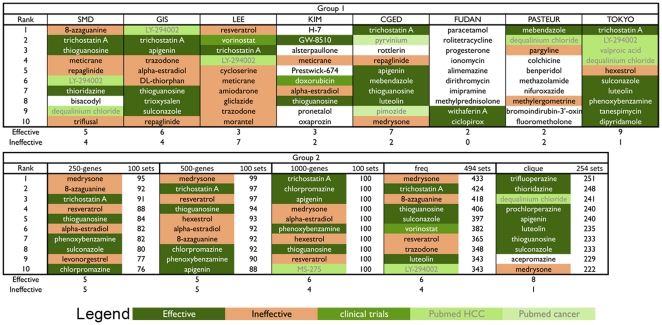
Effectiveness of drugs in Group 1 and Group 2. The top 10 drugs from each set were labeled according to their anti-cancer effects.

After conducting CMap analysis, the top 10 drugs from each set are listed (Figure 3). Some of the drugs, such as trichostatin A and thioguanosine, have also been reported in previous studies (Table 2), suggesting some degree of power for discovering potential drugs. In contrast, FUDAN and PASTUER shared very few common drugs with the other sets, a result of their only mild similarity in gene expression to the other sets (Figure S1). The disparity in drug predictions confirms the gene-set sensitivity of CMap. Therefore, to guarantee optimal drug discovery, several strategies were formulated to devise enriched gene sets.

### Gene expression of Group 2 (Derived EHCO2 sets)

#### a) Generation of Random Sets

With the collection of candidate HCC-related genes, a collection of possible combinations of simulated patient gene expression profiles was created to reflect the heterogeneous nature of HCC. Sets of 250 up-regulated and 250 down-regulated genes were selected randomly from the EHCO2 gene pools of up- and down-regulated sets, respectively, for a total of 100 sets.

Since a set of 500 genes comprises less than 15% of the total number of EHCO genes (4,020 genes), this might not be adequate to represent HCC. Selections of 500 up-regulated and 500 down-regulated genes and of 1,000 up-regulated and 1,000 down-regulated genes were also made for further comparison. A computer program written in Ruby that simulates the CMap calculations was implemented to handle the larger data inputs.

#### b) Generation of Frequent Sets

Since HCC is a heterogeneous disease, it is likely that genes reported in one study differ greatly from another study. Therefore, to simulate all possible scenarios, all combinations (n) of eight EHCO sets were created. In other words, any two studies (n = 2) were combined as a set, and this was continued until all studies were combined into a set (n = 8). In each combination, three frequency thresholds (k) were set (i.e, no genes were discarded when k = 1 and genes were kept if genes occurred in two or more studies when k = 2). A total of 1,021 sets were created.

#### c) Generation of Clique Sets

The notion of *clique* from the field of Graph Theory was utilized to enrich the gene sets. A protein-protein interaction network of EHCO2 genes was created, and cliques were extracted from this graph. A clique is a sub-graph where all the nodes are connected to each other. The simplest clique is the *3-clique*, 3 interconnected nodes, or a triangle. The proteins in the clique set might represent a possible protein complex, which is the preferred candidate for drug targeting [23]. Clique Analysis [21] was used to search for *3-clique* sets in each “Frequent set”.

### CMap analysis of Group 2 sets (Derived EHCO2 sets)

Group 2 containing five different HCC gene sets, including three “100-random sets”, the “Frequent sets”, and the “Clique sets”, was queried with CMap, and corresponding prioritized drug lists were generated (Figure 3).

### Using selected HCC gene signatures to reveal potential drugs with anti-proliferative or cytotoxic effects from CMap

Bioactive small molecules in CMap that reverse, at least in part, the HCC gene signatures may be drugs with potential to eradicate HCC cells. In fact, several identified drugs already have literature references of cancer studies. Drugs such as pyrvinium and levonorgestrel have PubMed references related to cancers, while MS-275 and LY-294002 are known to inhibit HCC cells. These drugs were marked as “PubMed Cancer” and “PubMed HCC”, respectively (Figure 3). Additionally, we selected the 50 top-occurring small molecules (Table S2) from each top 20 drugs of the 2 groups (a total prediction of 258 drugs) and determined the effects of these drugs on the proliferation of 4 HCC cell lines by MTT and/or clonogenic assays. Drugs with (1) an IC_50_ (concentration that inhibits cell growth by 50%) less than 10 µM or (2) a 50% reduction in number of colonies at 10 µM in the clonogenic assay were defined as effective against HCC cell lines. As shown in Table 2 and Figure S3, the viability of HCC cell lines was reduced by more than 50% after co-incubation with various concentrations of trichostatin A and tanespimycin for 72 hours (IC_50_<10 µM). These results were consistent with previous studies [24]–[27]. Drugs with IC_50_ over 10 µM (Table 2) were subjected to the clonogenic assay as a secondary screening. Treatment of Huh7 cells with 10 µM chlorpromazine and trifluoperazine dramatically reduced the clonogenic survival of Huh7 cells (Figure 3). In short, as shown in Table S2, 28 of the 50 top-ranked drugs were considered effective.

### Accuracy of drug prediction comparison

The effectiveness of the top 10 drugs from each set is depicted in Figure 2A. Group 1 sets showed great variability in prediction power, suggesting that gene signatures from a single study of a heterogeneous disease might not guarantee an optimal drug prediction result. We resorted to using combined studies for larger gene pools.

To this end, using EHCO's Confidence Set (Method S1) consisting of 1,821 up regulated and 1,477 down-regulated HCC-related genes, a randomization technique was employed to select gene signature sets of 250-gene, 500-gene, and 1,000-gene in order to represent the heterogeneous nature of HCC. As depicted in Figure 3, this method identified 13 drugs, 7 of which appeared in all three sets. The results were consistent despite the differences in gene number. Compared to the results of Group 1, however, this method did not significantly improve the accuracy or reduce the inaccuracy. Furthermore, several ineffective drugs even had higher rankings. The mediocre result can be explained by the lack of gene structure maintenance while randomly choosing genes.

Another gene selection strategy is to keep the most occurring genes, putting more emphasis on consensus genes and ignoring infrequent genes. To simulate studies, we generated all combinations of Group 1 sets and, for each set, set three occurrence thresholds (k). Under these stringent thresholds, some combinations did not have enough genes (probe sets <10) and were discarded, resulting in a total of 494 sets. As depicted in Figure 3, at first glance, the result was not as promising. However, once the result was stratified, as shown in Figure 2C, certain trends emerged. For all thresholds (k), sets with more studies (n) combined had better results. Furthermore, the accuracy (0.6) peaked when k = 2. In addition, as depicted in Figure S2B, while the unverified rate remained similar to sets of k = 1, sets of k = 2 had much lower ineffective rates. The above observations suggested an effective strategy to combine studies and enrich the gene sets using the frequency threshold.

While instituting a frequency threshold eliminates less occurring genes, gene sets can be further enriched by using Clique Analysis. This elimination, unlike that conducted in the Randomization sets, preserves the gene structures, making function-related genes sets. After eliminating sets with insufficient genes (probe sets <10), 254 sets were created. As depicted in Figure 3, the result reached 80%, and the previously highly ranked but ineffective drug medrysone was now ranked 10^th^. Furthermore, as presented in Figure 3C, the clique version of the sets outperformed the frequent versions in all cases. Among the 254 sets compared, there was an average of 86.22% and 91.71% reduction in up-regulated genes and down-regulated genes, respectively. Furthermore, 72% of the clique sets had a greater effective rate and 89% of the clique sets had a less ineffective rate compared to the corresponding frequent sets. The reduction in gene set numbers and improvement in accuracy strongly indicate that these selected genes play a vital role in HCC cells.

### Chlorpromazine and trifluoperazine decrease the growth of orthotopic liver tumors

**Figure 4:**
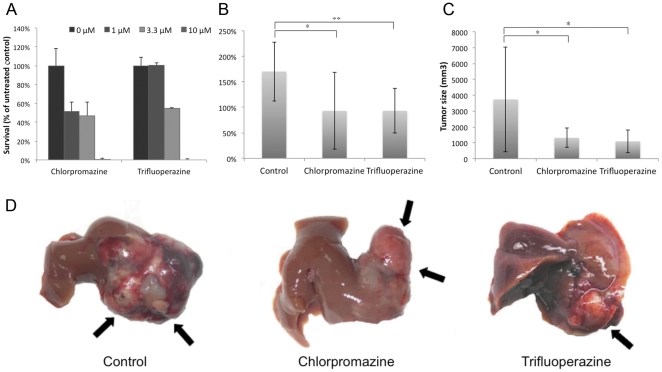
In-vitro and in-vivo effects of chlorpromazine and trifluoperazine. (**A**) **Chlorpromazine and trifluoperazine inhibit clonogenic survival.** Huh7 cells were incubated with chlorpromazine and trifluoperazine at various concentrations (1, 3.3, and 10 µM) for 15 days. Cell colonies were counted and expressed in terms of percent survival relative to the control. The data represent the mean±SD from three independent experiments. ***In vivo*** **effects of chlorpromazine and trifluoperazine on BNL cell orthotopic liver tumor models.** (B) The ratio of tumor size enlargement obtained from all animals in the therapeutic group after 21 days of treatment was calculated. (C) The sizes of the tumors obtained from the animals in the prophylactic group 21 days after tumor implantation were measured. *P<0.05, **P<0.005, compared to the control group. (D) The sizes of tumors obtained from the animals in the prophylactic group 21 days after tumor implantation were measured. Black arrows show the liver tumors.

To test for anti-tumor effects in a clinically relevant situation, we used an orthotopic liver tumor model representing a large tumor load. BNL cells (3×10^5^) were injected into the left liver lobe of an animal. Usually, a tumor nodule of ∼60–100 mm^3^ can be observed on day 14 after tumor implantation. Liver tumors were measured on day 35 using calipers. As shown in Figure 4, in the prophylaxis experiment, while animals in the control groups showed apparent tumor enlargement on day 35 compared to day 14 (170.2±58.0%), those treated with chlorpromazine showed less tumor enlargement (93.1±75.4%), and those treated with trifluoperazine also showed an inhibition of tumor enlargement (93.2±43.4%). The tumor growth in the chlorpromazine and trifluoperazine groups was significantly slower than that of the control group (chlorpromazine group vs. control group, *p*<0.05; trifluoperazine group vs. control group, *p*<0.005). For both treated groups, the therapeutic effects were obvious compared to the control group. In the therapeutic experiment, the tumor volume of the animals in the control groups was 3,739.6±3,304.0 mm^3^ on day 21, and those treated with the test agents showed decreased tumor growth (1,313.2±610.8 mm^3^ for chlorpromazine and 1,093.0±720.5 mm^3^ for trifluoperazine). The tumor growth in the chlorpromazine and trifluoperazine groups was significantly slower than that of the control group (chlorpromazine group vs. control group, p<0.05; trifluoperazine group vs. control group, p<0.05).

## Discussion

The ultimate goal of this study is to identify potential drug candidates for rapid screening for anti-HCC therapy via CMap. The heterogeneous nature of HCC yields gene signatures with little similarity; thus, it is a great challenge to formulate an optimal gene list for drug prioritization. To address this, we did the following: (1) collected HCC-related genes from various array sources and studies in the literature and organized EHCO2 as an updated public resource, (2) devised strategies for creating gene signatures from the heterogeneous data, (3) tested the CMap-predicted drugs *in vitro* and compared the performance of gene-selection strategies from the experiment, and (4) validated two candidate FDA-approved drugs, chlorpromazine and trifluoperazine, in orthotopic liver tumor models. This strategy provides a shortcut for the development of anti-HCC therapies. With an ever-increasing amount of genomic data available, other diseases of interest will also likely face similar difficulties. Our method provides a novel approach for combining several genomic studies, aiming for the precise prediction of drug efficacy.

Since the development of CMap, researchers have used this tool to investigate several cancers, including breast [28], colon [29], and prostate cancers [30], as well as nasopharyngeal carcinoma [31]. Using the EHCO2 datasets, we proposed to conduct CMap analysis with the aim of finding therapeutic drugs for HCC. The EHCO2 database provides us with diversified HCC samples for this purpose. In order to simulate possible combinations of HCC gene expression, we filtered genes by three different methods, including the “Frequent sets”, “Clique sets”, and randomly selected 100-member subsets of all occurring genes. The “Frequent sets” and “Clique sets” were better gene sets for precisely identifying potential drugs for HCC than the random sets. One plausible explanation is that in each of the random sets, unrelated genes were selected, and genes with high correlations might not be selected at once, thus compromising accuracy. On the other hand, the result of using the “Frequent Set” was far better than those of the individual sets, proving the usefulness of applying the “majority vote” method to the heterogeneous gene sets. Moreover, the use of “Clique Analysis” enriched the gene sets by only including genes with associated functions and resulting in higher accuracy (Figure 2C). One drawback is that the Clique Analysis reduces the gene number greatly, which is unsuitable for sets with limited numbers of genes. Clique Analysis performs well in the combination set study with a sufficient number of genes reported, yet performs poorly on single studies (data not shown). Finally, the comparison of the accuracy of predictions from all sets is listed in Figures 2B and 3. With a more concise gene signature, the CMap program generates a more precise prediction. In conclusion, the tight integration of CMap and EHCO2 data provides a good strategy for prioritizing drug selection from a chemical library.

Of the 50 prioritized drugs (Table S2) from each of the two groups, 28 were determined to have anti-tumor effects by the MTT and/or clonogenic assays. Some drugs (Table 2) were previously reported in PubMed for cancer treatment and even some for HCC, which verifies our results. Trichostatin A, which is a histone deacetylase inhibitor, may also inhibit tumor cell proliferation [25], [26], [32] (Figure S3A); Tanespimycin is a heat shock protein 90 (HSP90) inhibitor, which degrades HSP90 related client protein and induces tumor cell death [24] (Figure S3B). LY294002, which is a PI3K inhibitor, has been extensively studied to inhibit cancer cell growth by blocking the PI3K/Akt pathway [33]; Thioguanosine is a purine analog and an anti-tumor drug. It has been reported to inhibit ribosomal RNA maturation in hepatoma cells [27]. Interestingly, antipsychotic drugs such as chlorpromazine, trifluoperazine and thioridazine were also identified to have anti-tumor effects (Table 2).

Two drugs, chlorpromazine and trifluoperazine, were further tested for anti-tumor effects in an *in vivo* HCC study (Figure 4). They are both typical anti-psychotic drugs of the phenothiazine group. Chlorpromazine is also used to control intractable hiccup nausea and vomiting. Some studies have reported that chlorpromazine might inhibit tumor cell proliferation and cause cell-cycle arrest [34]–[36]. Trifluoperazine has also been demonstrated to induce cancer cell apoptosis [37]. Other potential therapeutic drugs are discussed in Method S1.

HCC is a heterogeneous and multi-factorial disorder that may involve distinct pathways in different individuals. Although the ideal strategy is to predict drugs for each individual or for patients with different etiologies (i.e., HBV, HCV, or alcohol-related) or clinical outcomes, to the best of our knowledge there are no proper paired sample arrays (diseased vs. normal liver tissues or cirrhotic liver tissue) that can be utilized to address this. Thus, the main limitation of this study is that the HCC datasets used for CMap construction are not uniformly associated with clinical outcomes or HCC etiologies. HCC behavior will vary in each individual patient and thus will result in varying risks for overall survival and tumor recurrence; drug efficacy may also vary as a result of this.

In summary, the shortage of new drugs for the treatment of HCC and the rapidly rising costs of drug development encourage efforts to explore methodology for drug-repurposing. Our bioinformatics analysis exploits the resources from EHCO2 and CMap to make connections between gene expression, disease, and drug action, resulting in the systematic identification of several potential therapeutic drugs. This finding in conjunction with future clinical trials may provide a paradigm of drug discovery for neglected diseases.

## Supporting Information

Method S1 **Supplementary method and discussion.**
(DOCX)

Figure S1 **Clustering Dendrogram for Group 1.**
(TIF)

Figure S2 **Results of the Frequent sets.** (A) Effective rate, (B) Ineffective rate, and (C) Unverified rate, of the top 10 drugs from each frequent set. *n* indicates numbers of studies combined while *k* indicates the frequency a gene should at least have to remain in the gene set.
(TIF)

Figure S3 **Trichostatin A and tanespimycin inhibit cell proliferation.** Each drug was administered at various concentrations (0.1 µM, 1 µM, and 10 µM) to 4 HCC cell lines, HepG2, PLC5, Mahlavu, and Huh7, for 72 hours. The cell viability was evaluated by the MTT assay. Trichostatin A (A) and tanespimycin (B) exhibited cytotoxicity effect. The data represent the mean±SD from three independent experiments.
(TIF)

Table S1 **Jaccard's Index between all Group 1 sets.** Jaccard's Index is used to measure the similarity between pairs of sets. The ratios in parentheses indicate the number of common genes out of the total number of genes for each pair.
(XLSX)

Table S2 **Potential 50 drugs identified from the Top 20 drugs from Group 1 and Group 2 sets.** Potential 50 drugs identified from the Top 20 drugs of all 13 sets.
(XLSX)
