# Supplementary material for: Gene Expression-Based Chemical Genomics Identifies Potential Therapeutic Drugs in Hepatocellular Carcinoma
Source: PLoS One. 2011 Nov 7;6(11):e27186. doi: 10.1371/journal.pone.0027186 (PMC3210146; doi:10.1371/journal.pone.0027186)
Supplement: Method S1 — Supplementary method and discussion. (DOCX) [file pone.0027186.s001.docx]

# Supplemntary Method

## Collection of HCC-related Gene Expression Signatures

We maintained and updated the eight original datasets in the first version of EHCO　[[1](#_ENREF_1)]. Some of the gene symbols and identifiers were corrected using the Gene Name Service [[2](#_ENREF_2)]. Some of the genes were excluded because they were discontinued from NCBI. PubMed, TableX_mRNA, and TableX_protein datasets were also updated with new genes. Briefly, for the PubMed dataset, we have extracted 1,084 genes (with gene names approved by HUGO Gene Nomenclature Committee) from approximately 4,500 abstracts in the PubMed category. Moreover, seven additional reports [[3-9](#_ENREF_3)] were manually added into the TableX_mRNA dataset. Similarly, four extra proteomics reports [[10-13](#_ENREF_10)] were included in the TableX_protein dataset. Among the HCC-related studies, EHCO2 further included six additional gene sets:

- UCSF [[14](#_ENREF_14)] used cDNA microarrays containing 17,000 unique human genes to analyze the gene expression profiles of 102 primary HCC and 74 non-tumor liver tissues. They identified 636 genes with official HUGO symbols that were highly expressed in HCC.
- CGED [[15](#_ENREF_15)] analyzed the gene expression profiles of 100 samples randomly selected from 120 HCC tissues, 86 non-tumor adjacent normal tissues and 32 normal liver tissues by adaptor-tagged competitive PCR (ATAC-PCR). Differential expression in normal and tumor tissues was observed for 596 of the 3,072 genes identified.
- FUDAN [[16](#_ENREF_16)] analyzed the gene expression profiles of hepatitis B virus-positive HCC through the generation of a large set of 5’-read expressed sequence tag (EST) clusters from HCC and non-cancerous liver samples by using cDNA microarrays. In addition, a commercial cDNA microarray was used for profiling gene expression. Taken together, these experiments identified 2,253 genes/ESTs with differential expression, resulting in a gene set of 493 genes with official HUGO symbols.
- PASTEUR [[17](#_ENREF_17)] applied cDNA microarrays to analyze the expression profiles of 15 cases of HCC. Genes with a ratio greater than or equal to 2 or a ratio less than 0.5 between tumor and non-tumor intensity were defined as up- or down-regulated, respectively. 84 genes with official HUGO symbols were defined in more than 30% of 30 comparisons of tumors versus non-tumors.
- TOKYO [[18](#_ENREF_18)] analyzed the gene expression patterns of 20 primary HCCs and their corresponding non-cancerous tissues by using a cDNA microarray consisting of 23,404 genes. When a signal intensity cutoff ratio of 2.0 (cancer versus non-cancer) was applied, 165 genes (including 69 ESTs) were up-regulated in 75% or more of the HCC samples examined. On the other hand, 170 genes (including 75 ESTs) were down-regulated in 65% or more of the case examined when a cutoff intensity ratio of 0.5 was applied. Together, 242 genes have official HUGO symbols.
- POFG [[19](#_ENREF_19)] used a computational method to identify 84 putative oncofetal genes (POFG) whose splicing pattern distribution is similar in fetal and tumorous adult tissues but different from or below detectable levels in normal adult tissue.

## Confident EHCO2 gene set

The integration of these data resulted in disagreement among different datasets, therefore, we selected 3,298 HCC-related genes (1,821 up-regulated and 1,477 down-regulated) as our confident set from the 4,020 HCC-related genes from EHCO2. The confident set consists of genes that can be distinguished by their expression as up-regulated or down-regulated in at least two-thirds of the datasets in which the gene is present. Those genes present in only one dataset are also included in the confident set.

## Calculation of a Similarity Matrix

To compare the similarity of the gene list between any pair of sets, Jaccard’s index was applied. The index between two lists is defined as the ratio of the number of intersecting items to the number of union items, or mathematically, *Jaccard(A,B) = (A and B)/(A or B).* Jaccard’s distance, or the dissimilarity, is defined as *1-Jaccard.* Jaccard’s distance matrix was used to perform hierarchical clustering using R.

# Supplementary discussion

Some other drugs, selected by CMap, had been reported to have anticancer effects. Dipyridamole, a platelet aggregation inhibitor, may induce cervical cancer cell apoptosis [[20](#_ENREF_20)]. Apigenin, a flavonoid, is present in fruits and vegetables and is believed to have an anticancer function [[21](#_ENREF_21)]. Studies of human malignant cancer cell lines have shown that apigenin inhibits cancer cell growth via the inhibition of cell proliferation, the promotion of cell cycle arrest, and apoptosis [[22](#_ENREF_22)]. We also identified some potential novel drugs for HCC, such as phenoxybenzamine and sulconazole. These drugs have never been reported to have anti-cancer effects in any previous study. The actual mechanism of these drugs still needs further investigation.

# Supplementary References

1. Hsu C-N, Lai J-M, Liu C-H, Tseng H-H, Lin C-Y, et al. (2007) Detection of the inferred interaction network in hepatocellular carcinoma from EHCO (Encyclopedia of Hepatocellular Carcinoma genes Online). BMC Bioinformatics 8: 66.

2. Lin K, Liu C, Chiou J, Tseng W, Lin K, et al. (2007) Gene name service: no-nonsense alias resolution service for Homo Sapiens genes. 2007 IEEE/WIC/ACM International Conferences on Web Intelligence and Intelligent Agent Technology Workshops: 185-188.

3. Ye QH, Qin LX, Forgues M, He P, Kim JW, et al. (2003) Predicting hepatitis B virus–positive metastatic hepatocellular carcinomas using gene expression profiling and supervised machine learning. Nature Medicine 9: 416-423.

4. Tsai CC, Chung YD, Lee HJ, Chang WH, Suzuku Y, et al. (2003) Large-scale sequencing analysis of the full-length cDNA library of human hepatocellular carcinoma. Journal of Biomedical Science 10: 636-643.

5. Tsai CC, Huang KW, Chen HF, Zhan BW, Lai YH, et al. (2006) Gene expression analysis of human hepatocellular carcinoma by using full-length cDNA library. Journal of Biomedical Science 13: 241-249.

6. Su WH, Chao CC, Yeh SH, Chen DS, Chen PJ, et al. (2007) OncoDB. HCC: an integrated oncogenomic database of hepatocellular carcinoma revealed aberrant cancer target genes and loci. Nucleic Acids Research 35: D727.

7. Nguyen H, Sankaran S, Dandekar S (2006) Hepatitis C virus core protein induces expression of genes regulating immune evasion and anti-apoptosis in hepatocytes. Virology 354: 58-68.

8. Iizuka N, Tsunedomi R, Tamesa T, Okada T, Sakamoto K, et al. (2006) Involvement of c-myc-regulated genes in hepatocellular carcinoma related to genotype-C hepatitis B virus. Journal of Cancer Research and Clinical Oncology 132: 473-481.

9. Iizuka N, Oka M, Yamada-Okabe H, Mori N, Tamesa T, et al. (2003) Differential gene expression in distinct virologic types of hepatocellular carcinoma: association with liver cirrhosis. Oncogene 22: 3007-3014.

10. Kim W, Lim O (2003) Comparison of Proteome between Hepatitis B Virus-and Hepatitis C Virus-Associated Hepatocellular Carcinoma. Clinical Cancer Research 9: 5493.

11. Li C, Hong Y, Tan YX, Zhou H, Ai JH, et al. (2004) Accurate Qualitative and Quantitative Proteomic Analysis of Clinical Hepatocellular Carcinoma Using Laser Capture Microdissection Coupled with Isotope-coded Affinity Tag and Two-dimensional Liquid Chromatography Mass Spectrometry*. Molecular & Cellular Proteomics 3: 399-409.

12. Liang CR, Leow CK, Neo JC, Tan GS, Lo SL, et al. (2005) Proteome analysis of human hepatocellular carcinoma tissues by two-dimensional difference gel electrophoresis and mass spectrometry. Proteomics 5: 2258-2271.

13. Yokoyama Y, Kuramitsu Y, Takashima M, Iizuka N, Toda T, et al. (2004) Proteomic profiling of proteins decreased in hepatocellular carcinoma from patients infected with hepatitis C virus. Proteomics 4: 2111-2116.

14. Patil M, Chua M, Pan K, Lin R, Lih C, et al. (2005) An integrated data analysis approach to characterize genes highly expressed in hepatocellular carcinoma. Oncogene 24: 3737-3747.

15. Kato K, Yamashita R, Matoba R, Monden M, Noguchi S, et al. (2005) Cancer gene expression database (CGED): a database for gene expression profiling with accompanying clinical information of human cancer tissues. Nucleic acids research 33: D533.

16. Xu X, Huang J, Xu Z, Qian B, Zhu Z, et al. (2001) Insight into hepatocellular carcinogenesis at transcriptome level by comparing gene expression profiles of hepatocellular carcinoma with those of corresponding noncancerous liver. Proceedings of the National Academy of Sciences 98: 15089.

17. Delpuech O, Trabut J, Carnot F, Feuillard J, Brechot C, et al. (2002) Identification, using cDNA macroarray analysis, of distinct gene expression profiles associated with pathological and virological features of hepatocellular carcinoma. Oncogene(Basingstoke) 21: 2926-2937.

18. Okabe H, Satoh S, Kato T, Kitahara O, Yanagawa R, et al. (2001) Genome-wide analysis of gene expression in human hepatocellular carcinomas using cDNA microarray: identification of genes involved in viral carcinogenesis and tumor progression. Cancer Research 61: 2129.

19. Liu C-H, Lin K-T, Huang C-YF, Shann Y-J, Lin Y-S, et al. (2011) Genome-Wide Detection of Putative Oncofetal Genes in Human Hepatocellular Carcinoma by Splicing Pattern Comparison. iConcept Transaction on Computational Intelligence in Bioinformatics (TCIB).

20. Chen WH, Yin HL, Chang YY, Lan MY, Hsu HY, et al. (1997) Antiplatelet drugs induce apoptosis in cultured cancer cells. Kaohsiung J Med Sci 13: 589-597.

21. Singh JP, Selvendiran K, Banu SM, Padmavathi R, Sakthisekaran D (2004) Protective role of Apigenin on the status of lipid peroxidation and antioxidant defense against hepatocarcinogenesis in Wistar albino rats. Phytomedicine 11: 309-314.

22. Choi EJ, Kim GH (2009) Apigenin Induces Apoptosis through a Mitochondria/Caspase-Pathway in Human Breast Cancer MDA-MB-453 Cells. J Clin Biochem Nutr 44: 260-265.
