# Supplementary figures and images for: Gene Expression-Based Chemical Genomics Identifies Potential Therapeutic Drugs in Hepatocellular Carcinoma
Source: PLoS One. 2011 Nov 7;6(11):e27186. doi: 10.1371/journal.pone.0027186 (PMC3210146; doi:10.1371/journal.pone.0027186)

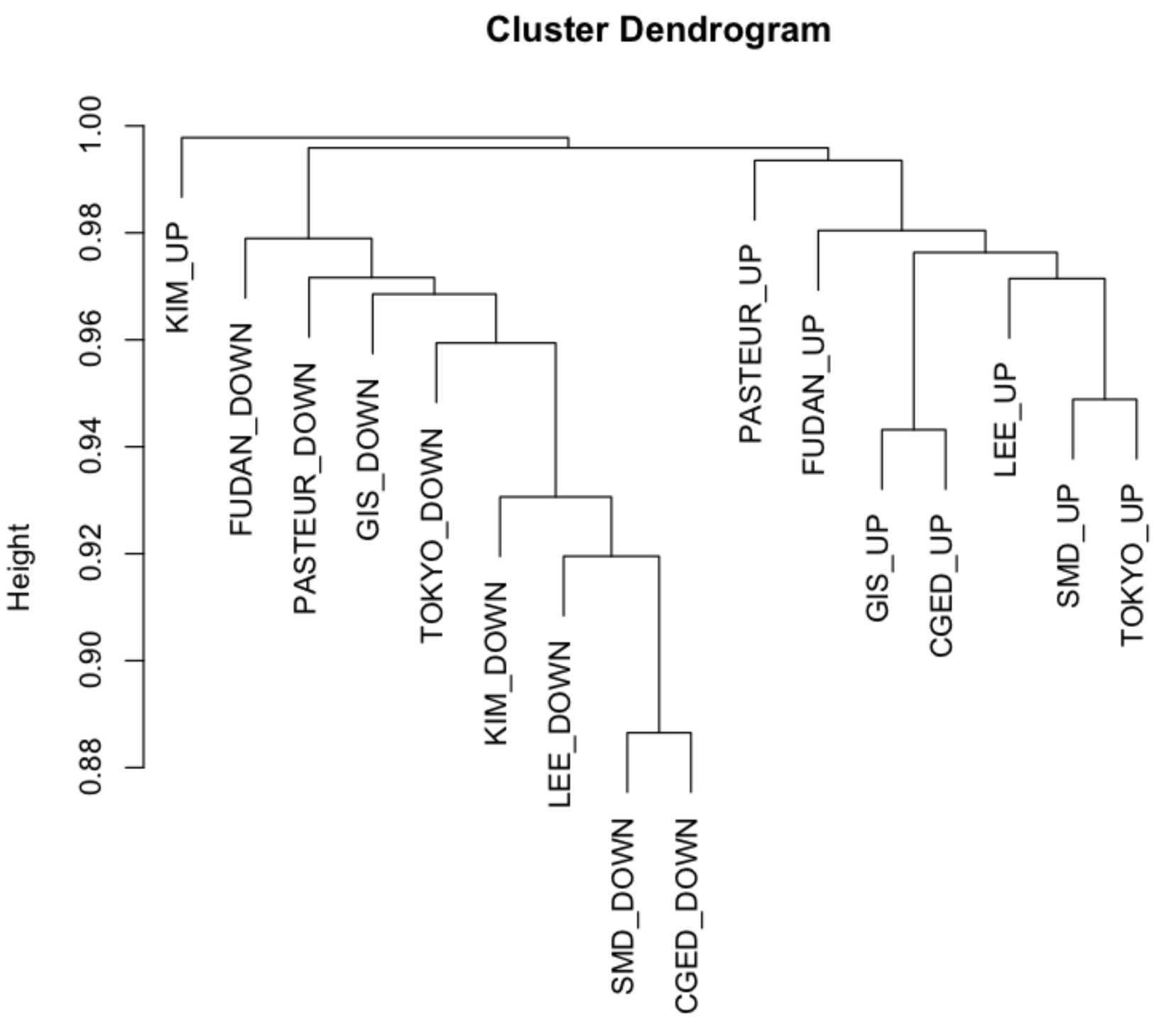

Supplement: Figure S1 — Clustering Dendrogram for Group 1. (TIF) [file pone.0027186.s002.tif]

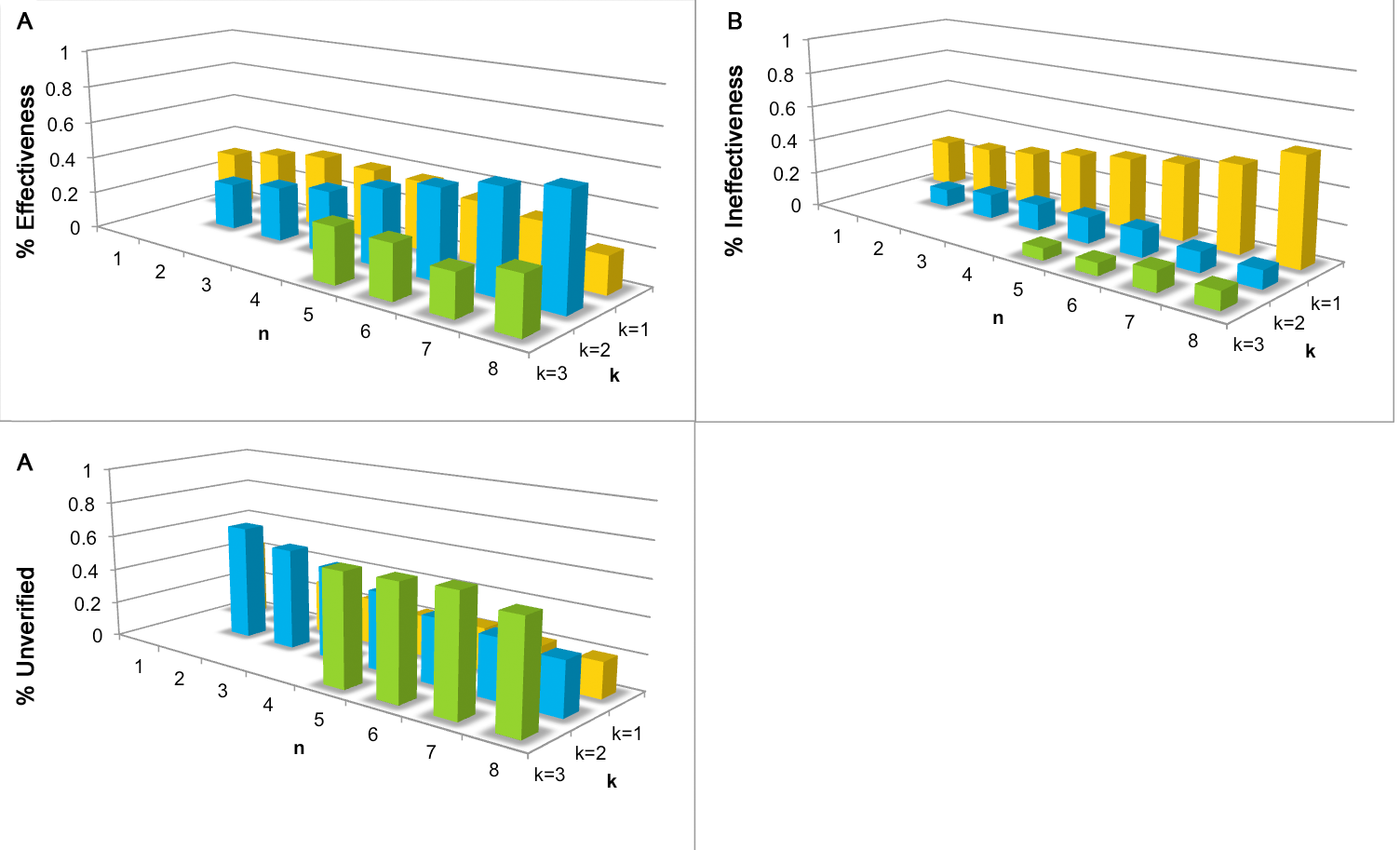

Supplement: Figure S2 — Results of the Frequent sets. (A) Effective rate, (B) Ineffective rate, and (C) Unverified rate, of the top 10 drugs from each frequent set. n indicates numbers of studies combined while k indicates the frequency a gene should at least have to remain in the gene set. (TIF) [file pone.0027186.s003.tif]

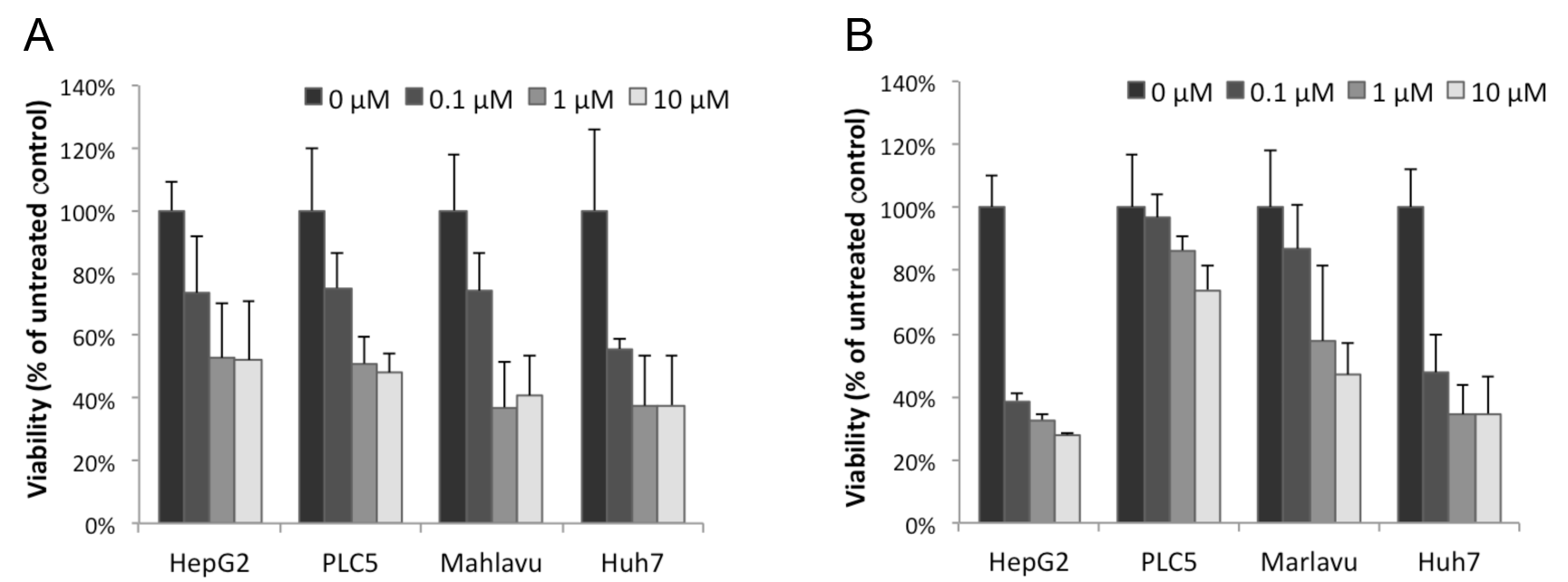

Supplement: Figure S3 — Trichostatin A and tanespimycin inhibit cell proliferation. Each drug was administered at various concentrations (0.1 µM, 1 µM, and 10 µM) to 4 HCC cell lines, HepG2, PLC5, Mahlavu, and Huh7, for 72 hours. The cell viability was evaluated by the MTT assay. Trichostatin A (A) and tanespimycin (B) exhibited cytotoxicity effect. The data represent the mean±SD from three independent experiments. (TIF) [file pone.0027186.s004.tif]
